# NK cell immunesenescence is increased by psychological but not physical stress in older adults associated with raised cortisol and reduced perforin expression

**DOI:** 10.1007/s11357-015-9748-2

**Published:** 2015-02-07

**Authors:** Niharika Arora Duggal, Jane Upton, Anna C. Phillips, Peter Hampson, Janet M. Lord

**Affiliations:** 1 MRC-Arthritis Research UK Centre for Musculoskeletal Ageing Research, School of Immunity and Infection, University of Birmingham, Birmingham, B15 2TT UK; 2 School of Sport and Exercise Sciences, University of Birmingham, Birmingham, B15 2TT UK

**Keywords:** NK cell, Stress, Immunesenescence, Cortisol

## Abstract

NK cell cytotoxicity (NKCC) reduces with age and this has been associated previously with increased mortality. The immune response is also modulated by stress, and here, we assessed the effect of the physical stress of hip fracture and the psychological stress of depression on NKCC in an aged immune system. NKCC was assessed in 101 hip fracture patients (81 female) 6 weeks and 6 months after injury and in 50 healthy age-matched controls (28 female). Thirty-eight patients were depressed at 6 weeks post-injury, and NKCC was reduced in patients who developed depression compared with non-depressed hip fracture patients (*p* = 0.004) or controls (*p* < 0.02). NKCC remained lower in the depressed patients compared to those without depression 6 months post-fracture (*p* = 0.017). We found reduced expression of perforin in NK cells of depressed hip fracture patients compared with controls at 6 weeks (*p* = 0.001) post-fracture. Serum cortisol levels were also elevated in patients with depression compared to non-depressed patients at 6 weeks (*p* = 0.01) and 6 months (*p* = 0.05). NK cells treated with dexamethasone showed a concentration-dependent reduction in NKCC and perforin expression. We propose that depression is the major factor affecting NK cell immunity after hip fracture.

## Introduction

Hip fracture is one of the most frequent physical traumas in older adults with an estimated 370,000 over 65 year olds falling every year in the UK alone, and the resulting number of hip fractures is predicted to rise to 120,000 by 2015 (Johnell et al. 1992). Even though hip fracture is treatable, it has a devastating effect on the life of older individuals and has been associated with poor outcomes: one in four patients die within a year of their fracture (Haentjens et al. 2007) and less than 30 % of patients return to their previous level of functioning (Jette et al. 1987).

Ageing is also accompanied by psychological distress and behavioural alterations; for instance, a high prevalence of depression has been reported in older adults (Ritchie et al. 2004). In addition, physical stress is a frequent precipitator of depression and a high rate of depression ranging from 9 to 47 % has been reported in older hip fracture patients (Holmes and House 2000). Depression in this patient population has a substantial effect on their post-fracture survival (Nightingale et al. 2001) and functional outcome (Holmes and House 2000).

It is now well established that a decline in immune function occurs with age, termed immunesenescence (Meyer 2005; Panda et al. 2009), resulting in an increased risk of infections, reduced vaccination responses and chronic illnesses (Castle 2000; Meyer 2005). Immunesenescence is evident in both the adaptive and innate arms of the immune system and several groups, including our own, have reported a decline in the function of NK cells (Di Lorenzo et al. 1999; Hazeldine et al. 2012). These cells play a key role in anti-viral and anti-cancer immunity, and although with age the numbers of these cells increase, their cytotoxic function declines (Almeida-Oliveira et al. 2011; Sansoni et al. 1993). NK cells kill virus-infected cells and cancer cells via ligation of death receptors and also by release of a pore-forming agent perforin and the apoptosis-inducing molecule granzyme (Smyth et al. 2005). Perforin creates pores in the target cell membrane allowing entry of granzyme which then induces caspase-dependent apoptosis (Law et al. 2010; Trapani and Smyth 2002). We have shown recently that the reduced cytotoxicity with age is due to reduced release of perforin from NK cells (Hazeldine et al. 2012).

The immune system does not operate in isolation, and a range of chronic stressors such as bereavement (Phillips et al. 2006), relationship stress (Kiecolt-Glaser et al. 2005), caregiving (Kiecolt-Glaser et al. 1996) and depression (Zorrilla et al. 2001) have been shown to suppress immune responses. In particular, the work of Kiecolt-Glaser and colleagues has shown that NK cells are susceptible to stress, with reduced NK cell cytotoxicity seen in caregivers (Esterling et al. 1996). Psychological stress may therefore exacerbate the suppressive effect of age on immune functioning, such that older individuals experiencing chronic stress are less able to mount successful immune responses (Graham et al. 2006). This is supported by work in stressed aged mice which have been shown to have reduced NK cell activity and lower survival compared to young stressed mice (Padgett et al. 1998). Chronic stress-associated immune suppression in older adults has clinical consequences, including an increased risk of bacterial and urinary infections in older adults post-surgery compared to young patients matched for clinical trauma level (Butcher et al. 2003).

A hallmark of the neuroendocrine immune response to stress is the activation of the hypothalamus-pituitary-adrenal (HPA) axis (Smith and Vale 2006) leading to increased circulating levels of immune suppressive glucocorticoids (Tsigos and Chrousos 2002). Glucocorticoids such as cortisol suppress the activity of many immune cells, including NK cells (Krukowski et al. 2011). Dehydroepiandrosterone sulphate (DHEAS), which is also produced by the adrenal gland, has been reported to have anti-depressive, anti-glucocorticoid and immune-enhancing properties (Hazeldine et al. 2010). Ageing is accompanied by changes to the HPA axis termed the adrenopause, associated with reduced DHEAS (Orentreich et al. 1984; Parker et al. 1997) and unaltered or slightly raised levels of cortisol (Pedersen et al. 2001). This results in an age-associated heightened HPA axis response with elevation in the cortisol/DHEAS ratio which could contribute to further immune dysregulation in older adults (Butcher et al. 2005).

In this study, we hypothesised that psychological stress, specifically depression, would act additively with the physical stress of hip fracture to amplify the effect of ageing upon immunity (immunesenescence), with specific reference to NK cell cytotoxicity.

## Results

### Depressive symptoms in patients and controls

The full demographic statistics for the participants in the study have been reported previously (Phillips et al. 2013). The key data for this study relate to the level of depressive symptomatology. Depressive symptoms in hip fracture patients and controls were measured using the Geriatric Depression Scale. Four to 6 weeks after their hip fracture, 38 of the 101 hip fracture patients recruited had GDS scores of 6 or more, indicative of depression. None of the age-matched healthy controls had scores of 6 or more. Patients were classified into two groups on the basis of their GDS scores: hip fracture patients with a GDS score of 5 or less were classified as non-depressed (HF; hip fracture only), those with a score of greater than 6 or more were categorised as depressed (HF + D; hip fracture patients with depression). The healthy controls were slightly younger (74.9 ± 5.63) than the hip fracture group without depressive symptoms (83.9 ± 7.48) and hip fracture group with depressive symptoms (84.0 ± 8.62), and as a result, all data were adjusted for age in the statistical analyses.

### The composition of the circulating NK cell pool in hip fracture patients

There were no significant differences in the percentage of NK cells in the PBMC pool *F*(2, 108) = .95, *p* = 0.38, *η* ^2^ = .01, or the absolute numbers of NK cells *F*(2, 55) = .47, *p* = 0.62, *η* ^2^ = .01 (Table 1) between the three subject groups. Further, on examining the distribution of the two NK cell subsets—CD56^dim^ and CD56^bright^—we found no difference in the percentage of CD56^dim^ NK cells, *F*(2, 133) = 2.52, *p* = 0.08, *η* ^2^ = .03, or of CD56^bright^ NK cells, *F*(2, 133) = 1.84, *p* = 0.16, *η* ^2^ = .02 between hip fracture patients with and without depression and healthy controls (Table 1). Therefore, the CD56^dim/bright^ ratio also did not differ between the three subject groups, *F*(2, 133) = 1.95, *p* = 0.14, *η* ^2^ = .03 (Table 1).

**Table 1:** NK cell subsets in hip fracture patients 6 weeks post-fracture

Mean (SD)				
Variable	Controls	Hip fracture patients (HF)	Hip fracture patients with depressive symptoms (HF + D)	*p* value
NK cells (% of PBMCs)	12.74 (7.58)	14.66 (6.99)	15.32 (8.70)	.32
NK cell absolute numbers (10^9^/ml)	.18 (.08)	.17 (.09)	.26 (.24)	.14
CD56^dim^ NK (% of PBMCs)	14.32 (6.68)	14.70 (7.57)	13.41 (7.12)	.76
CD56^dim^ NK cell numbers (10^9^/ml)	.18 (.08)	.18 (.08)	.19 (.15)	.94.
CD56^bright^ NK (% of PBMCs)	.67 (.44)	.58 (.40)	.59 (.37)	.63
CD56^bright^ NK cell numbers ( 10^9^/ml)	.01 (.02)	.006 (.003)	.01 (.01)	.44
CD56 ^dim/bright^ ratio	45.43 (28.31)	34.37 (29.06)	39.81 (24.94)	.52

CD57 expression on NK cells is suggestive of a mature terminally differentiated phenotype with decreased proliferation and reduced responsiveness to cytokines (Lopez-Verges et al. 2010). The percentage of NK cells expressing CD57 *F*(2, 54) = 1.31, *p* = 0.27, *η* ^2^ = .04 and the CD57 expression levels *F*(2, 54) = .12, *p* = 0.88, *η* ^2^ = .004 also did not differ between the three groups (Table 2). Next, we evaluated the expression of CD16 on NK cells of hip fracture patients as an indication of their potential to carry out antibody-dependent cell cytotoxicity (ADCC). The percentage of NK cells expressing CD16 *F*(2, 44) = 1.06, *p* = 0.35, *η* ^2^ = .04 and the expression levels *F*(2, 44) = .59, *p* = 0.55, *η* ^2^ = .02 did not differ between the three groups (Table 2). A range of activation receptors expressed by NK cells are involved in recognising ligands on tumour cells and have been associated with NK cell cytotoxicity (Moretta et al. 2006). Evaluation of the expression of activating receptor NKG2D on NK cells revealed that the percentage of NK cells expressing NKG2D *F*(2, 42) = .67, *p* = 0.51, *η* ^2^ = .03 and the expression levels *F*(2, 42) = .97, *p* = 0.38, *η* ^2^ = .04 did not differ between the three groups (Table 2).

**Table 2:** NK cell phenotypic characterisation in patients 6 weeks post-hip fracture

Mean (SD)				
Variable	Controls	Hip fracture patients (HF)	Hip fracture patients with depressive symptoms (HF + D)	*p* value
CD57^+ve^ NK cells (% of NK cells)	52.17 (13.46)	49.79 (16.53)	57.59 (14.90)	.27
CD57 expression (MFI)	166.56 (81.68)	163.74 (85.49)	191.91 (117.96)	.60
CD16^+ve^ NK (% of NK cells)	79.52 (13.97)	85.34 (9.81)	85.76 (13.91)	.34
CD16 expression (MFI)	201.56 (44.37)	207.02 (70.95)	225.70 (96.96)	.65
NKG2D^+ve^ NK (% of NK cells)	91.53 (4.66)	93.47 (5.78)	92.70 (4.32)	.47
NKG2D expression (MFI)	25.70 (4.11)	29.32 (5.78)	37.84 (40.10)	.36

### NK cell cytotoxicity in hip fracture patients

**Fig. 1:**
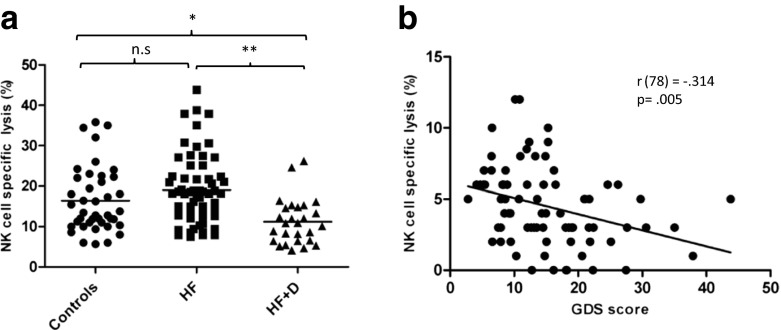
NK cell cytotoxicity in hip fracture patients. **a** Flow cytometry analysis of NKCC and conjugate formation by post-culture immunostaining of effector and target co-cultures with CD56-PE for identification of NK cells and K562 cells. **b** NKCC of healthy elderly controls and hip fracture patients with and without depression (healthy controls: *n* = 34; hip fracture patients without depression (*HF*): *n* = 38; hip fracture patients with depression (*HF+D*): *n* = 26). The mean value is indicated by the *bar*. *p < 0.005; **p < 0.001. **c** Correlation between GDS depression scores and NK cell cytotoxicity

The major mechanism utilised by NK cells to mediate protection against virally infected or tumour cells is via direct cell lysis of target cells. We found that NKCC towards K562 target cells differed between the groups, *F*(2, 114) = 5.55, *p* = 0.004, *η* ^2^ = .08 (Fig. 1b), but the impairment was restricted to the hip fracture patients who developed depression, *p* = 0.01, compared to controls, and *p* = 0.004, compared to hip fracture alone. When all of the above analyses were repeated with adjustment for age, the results remained the same (data not shown). Further, we found an association between NK cell cytotoxicity and depressive symptoms in hip fracture patients, *r*(78) = −.355, *p* = 0.001 (Fig. 1c), suggesting that the higher the GDS (depression) score, the lower the NK cell cytotoxicity.

### Conjugate formation and expression of cytotoxic proteins by NK cells in hip fracture patients

**Fig. 2:**
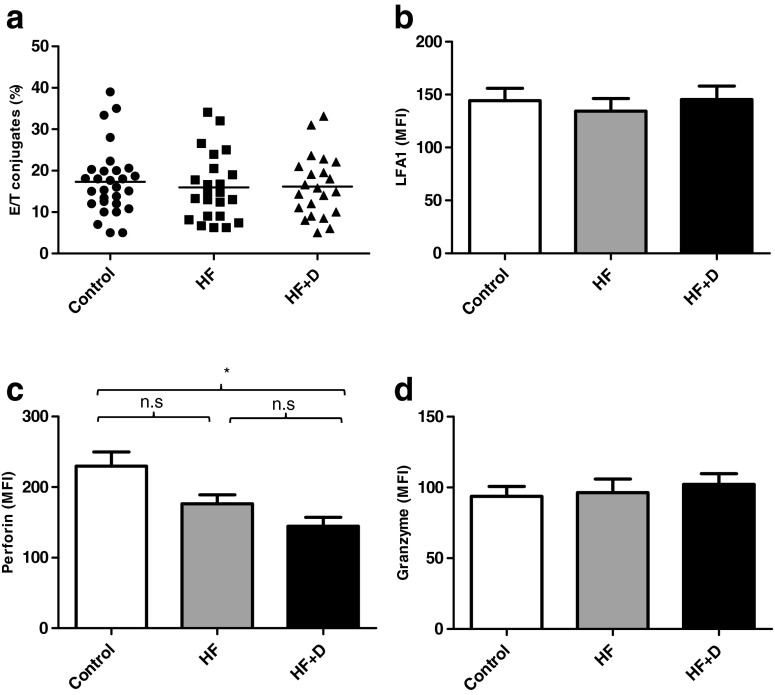
Conjugate formation and cytotoxic protein expression in hip fracture patients. **a** NK cell and target cell conjugate formation of healthy elderly controls and hip fracture patients with and without depression (healthy controls: *n* = 28; hip fracture patients without depression (*HF*): *n* = 22; hip fracture patients with depression (*HF+D*): *n* = 22). The mean value is indicated by the *bar*. **b** Surface expression of adhesion molecule LFA-1 on NK cells of healthy elderly controls (*n* = 15), hip fracture patients without depression (*n* = 15) and depressed hip fracture patients (*n* = 15). **c** Perforin expression by NK cells from hip fracture patients with depression (*n* = 26), hip fracture patients without depression (*n* = 26) and healthy controls (*n* = 26). **d** Granzyme B expression by NK cells from hip fracture patients with depression (*n* = 20), hip fracture patients without depression (*n* = 20) and healthy controls (*n* = 20). *p < 0.001

The induction of NK cell cytotoxicity is dependent on cell contact between NK cells and target cells to ensure precise targeting of the cytolytic protein perforin to the target cell (Orange 2008). In this study, we did not find any significant differences in the ability of NK cells to form conjugates with K562 target cells between the healthy controls and hip fracture patients with and without depression *F*(2, 72) = .41, *p* = 0.66, *η* ^2^ = .01 (Fig. 2a). Cell conjugate formation is a dynamic process involving adhesion molecules, and engagement of lymphocyte function-associated antigen 1 (LFA-1) by its ligand ICAM-1 on target cells is a central step in stable adhesion of NK cells to their target cells and is essential for NK cell cytotoxicity (Matsumoto et al. 2000). On examining LFA1 expression between the three groups, we also found no difference between groups *F*(2, 37) = .15, *p* = 0.85, *η* ^2^ = .009 (Fig. 2b); further supporting the conclusion that impaired NK cell cytotoxicity of depressed hip fracture patients was not a result of reduced conjugate formation.

Perforin and granzyme are the main constituents of cytotoxic granules that are responsible for inducing target cell death. Perforin is a multidomain protein that oligomerises to form pores in the target cell membrane facilitating delivery of granzymes into the cytosol of target cells (Law et al. 2010). Immunostaining to measure perforin expression by NK cells revealed significant differences between the three groups, *F*(2, 75) = 7.89, *p* = 0.001, *η* ^2^ = .17, with the reduction in perforin expression restricted to the hip fracture patients with new onset depression compared to controls, *p* < 0.001 (Fig. 2c). Even though perforin expression on NK cells of hip fracture patients without depressive symptoms was lower than healthy controls, this difference was not statistically significant, *p* = 0.16. Granzymes are serine proteases responsible for triggering target cell death via direct caspase activation and triggering mitochondrial permeabilisation (Cullen and Martin 2008). On examining intracellular granzyme B expression by NK cells, we did not observe any significant differences in expression levels in NK cells, *F*(2, 55) = .16, *p* = 0.84, *η* ^2^ = .006 (Fig. 2d) between the three groups.

### Serum cortisol and DHEAS levels in hip fracture patients

**Fig. 3:**
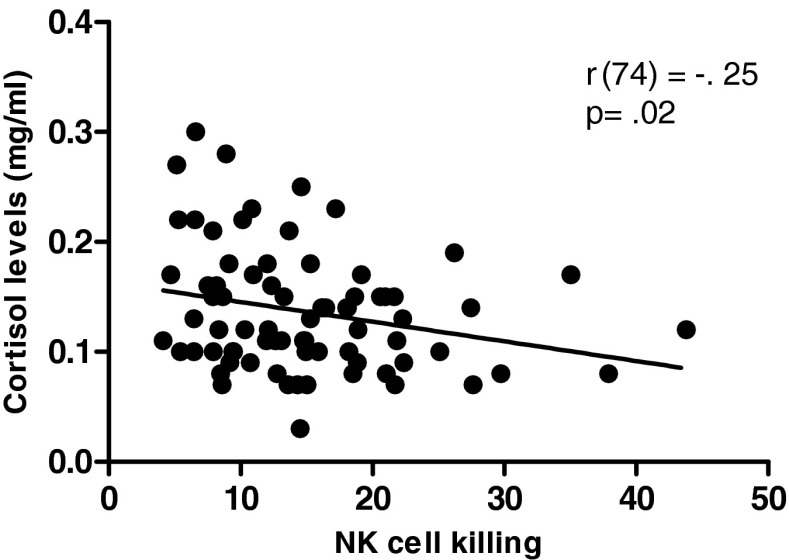
Relationship of serum cortisol to NKCC. Serum cortisol for healthy controls (*n* = 34) and hip fracture patients (*n* = 64) were correlated with values for NKCC

Analysis of cortisol levels after 6 weeks revealed significantly higher serum cortisol levels in hip fracture patients with depressive symptoms (0.17 ± 0.05 mg/ml) compared with healthy controls (0.13 ± 0.04 mg/ml; *p* = 0.006) or with hip fracture patients without depression (0.12 ± 0.04 mg/ml; *p* < 0.001). By 6 months, the cortisol levels were still significantly raised in the depressed group (0.14 ± 0.04 mg/ml) compared with hip fracture patients without depression (0.11 ± 0.03 mg/ml; *p* < 0.01). Further, a significant association was found between serum cortisol levels and NKCC in hip fracture patients, *β* = −.25, *p* = 0.02, Δ*R* ^2^ = .06 (Fig. 3), but not with NK cell perforin expression (*p* = 0.76). DHEAS levels were also significantly lower after 6 weeks in hip fracture patients with depressive symptoms (0.17 ± 0.14 mg/ml) compared with healthy controls (0.81 ± 0.36 mg/ml; *p* < 0.001) and the hip fracture alone group (0.43 ± 0.80 mg/ml; *p* = 0.008). Also, DHEAS levels of hip fracture patients without depression were significantly lower than healthy controls (*p* = 0.03). However, serum DHEAS did not relate to NKCC (*p* = 0.58) or perforin expression (*p* = 0.30). Also by 6 months after injury serum, DHEAS levels did not significantly differ between hip fracture patients with (0.23 ± 0.22 mg/ml) and without (0.27 ± 0.20 mg/ml) depressive symptoms (*p* = 0.14).

### Testing for mediation of depression effects on NK cell function by cortisol

In order to test whether the group differences in cortisol were driving the group differences in NKCC, a series of linear regression models were run. Age was entered into the model at block 1 as a covariate followed by group (depressed hip fracture, non-depressed hip fracture, healthy controls) with NKCC as the dependent variable. As expected, group significantly predicted NKCC, *β* = −.27, *p* = 0.01, Δ*R* ^2^ = .06, such that the lowest killing ability was observed among the depressed group as previously. On its own, cortisol also predicted NKCC, *β* = −.23, *p* = 0.02, Δ*R* ^2^ = .05, as expected. However, when cortisol was also entered into the model at step 2 as a potential mediator, the association between group and NKCC remained significant, *β* = −.23, *p* = 0.03, Δ*R* ^2^ = .04, suggesting that cortisol was not directly mediating the link between participant group and NKCC, though it could contribute separately to depression and reduced NKCC after hip fracture.

### Treatment with dexamethasone suppresses NK cell killing in vitro

**Fig. 4:**
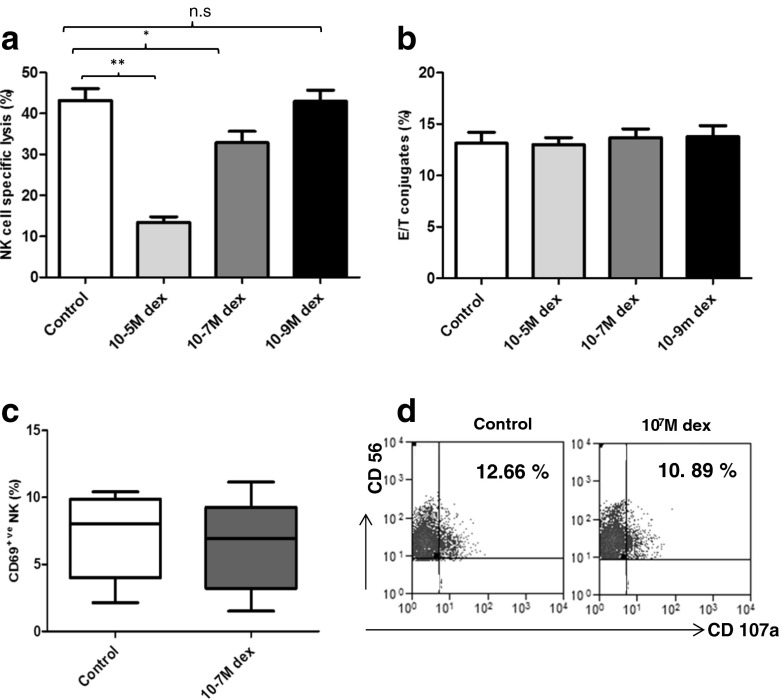
Effect of in vitro dexamethasone treatment on NK cell cytotoxicity. NK cells were treated with varying concentrations of dexamethasone for 16 h. **a** NKCC or **b** NK cell-target cell conjugate formation values for healthy donors (*n* = 9) treated with dexamethasone (10^−5^, 10^−7^ and 10^−9^ M) are shown. Data are mean ± SEM. *p < 0.05; **p < 0.001. **c** The percentage of CD69^+^ NK cells in dexamethasone treated and untreated cells (control) for *n* = 5 donors. **d** Flow cytometric plots of the percentage of CD3^−ve^CD56^+ve^ NK cells expressing CD107a following stimulation with K562 cells, in the absence of presence of dexamethasone (10^7^ M)

Glucocorticoids are known to exert a suppressive effect on NK cell killing (Holbrook et al. 1983; Krukowski et al. 2011). As we found increased serum cortisol levels correlated with reduced NKCC in the cohort of patients studied here, the possible involvement of cortisol was investigated further (Duggal et al. 2013). We performed an in vitro incubation of NK cells with dexamethasone (a synthetic glucocorticoid), for 18 h prior to an NKCC assay with K562 cells. Dexamethasone treatment resulted in a dose-dependent reduction in NK cell lysis of K562 cells (Fig. 4a) at 10^−5^ M dexamethasone (*p* < 0.001) and 10^−7^ M (*p* < 0.04) compared to untreated NK cells, but not at 10^−9^ M (*p* = 0.90). 10^−7^ M dexamethasone is equivalent to the physiological serum cortisol levels observed in hip fracture patients and has been referred to as a physiological concentration of dexamethasone in previous studies (Bush et al. 2012; Krukowski et al. 2011).

To exclude the possibility that suppressed NK cell killing seen at 10^−7^ M dexamethasone was caused by increased apoptosis of NK cells, we stained NK cells incubated for 18 h for Annexin V and sytox blue. There was no significant difference in the percentage of Annexin V-positive NK cells between untreated cells and NK cells treated with 10^−7^ M dexamethasone, *F*(1, 8) = .09, *p* = 0.77, *η* ^2^ = .01 (data not shown).

**Fig. 5:**
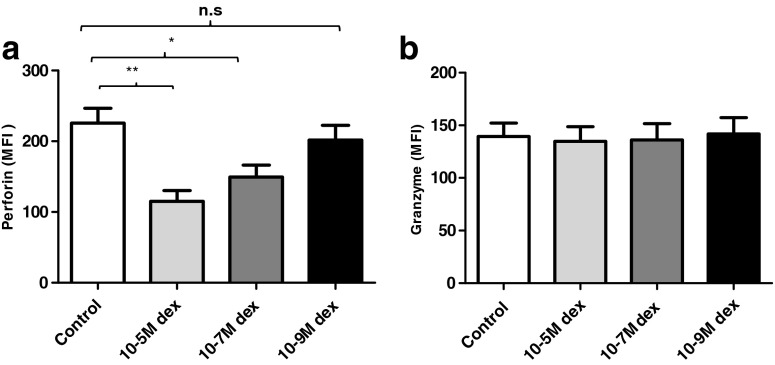
Perforin and granzyme B expression by NK cells post-dexamethasone treatment. **a** Perforin expression (MFI) by NK cells from young donors upon treatment with dexamethasone at the concentration shown. Data are mean ± SEM (*n* = 8). **b** Granzyme B expression (MFI) by NK cells on treatment with dexamethasone at the concentrations shown. Data are mean ± SEM (*n* = 5). *p < 0.05; **p < 0.001

On analysing the ability of NK cells to form conjugates with K562 target cell after dexamethasone treatment, we found no significant differences in the ability of NK cells to form conjugates, *F*(3, 24) = .15, *p* = 0.92, *η* ^2^ = .02 (Fig. 4b). We also found no effect of dexamthasone on CD69 expression, *F*(1, 8) = .12, *p* = 0.72, *η* ^2^ = .01 (Fig. 4c), or induction of CD107a, *F*(3, 24) = .85, *p* = 0.47, *η* ^2^ = .09 (Fig. 4d). In contrast, when we examined perforin expression by NK cells following dexamethasone treatment, we found that the expression of perforin was significantly reduced by treatment with 10^−5^ M dexamethasone (*p* = 0.001) and 10^−7^ M (*p* > 0.04) (Fig. 5a). Additionally, granzyme B expression levels were also measured on NK cells after dexamethasone treatment, but we did not observe any significant differences in the percentage of NK cells expressing granzyme B, *F*(3, 16) = .03, *p* = 0.99, *η* ^2^ = .007 (data not shown), or the granzyme expression levels, *F*(3, 16) = .03, *p* = 0.99, *η* ^2^ = .006 (Fig. 5b).

### Recovery of NK cell cytotoxicity in hip fracture patients 6 months after surgery

**Fig. 6:**
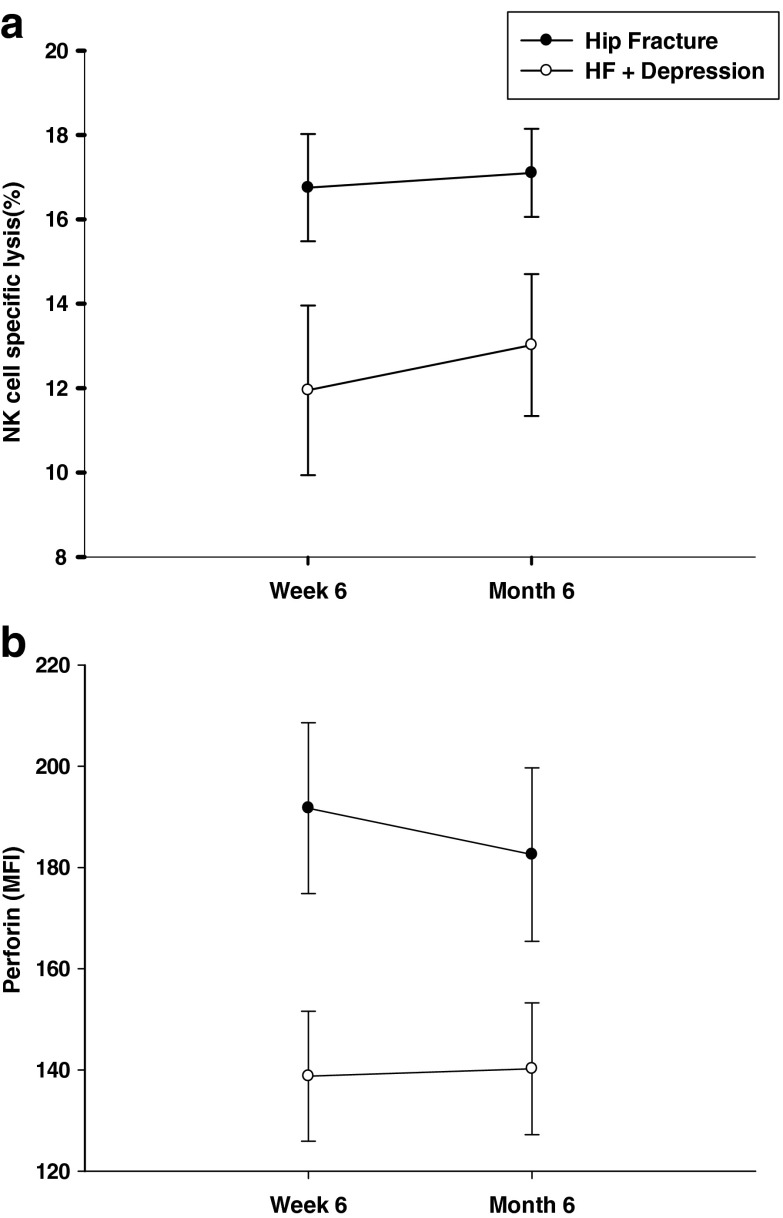
NKCC and NK cell perforin expression in hip fracture patients 6 weeks and 6 months post-surgery. **a** NKCC and **b** perforin expression (MFI) by NK cells in hip fracture patients with and without depressive symptoms 6 weeks and 6 months post-surgery. **a** NK cell specific lysis for hip fracture patients with depressive symptoms (HF + D; *n* = 12) and without depressive symptoms (HF; *n* = 30) at 6 weeks and 6 months post-injury. **b** Perforin expression in NK cells in hip fracture patients with depressive symptoms (HF + D; *n* = 19) and without depressive symptoms (HF; *n* = 11) at 6 weeks and 6 months post-injury. Data are mean ± SEM

On comparing NK cell cytotoxicity in hip fracture patients with and without depression at 4–6 weeks and 6 months post-surgery, there was no significant effect of time, *F*(1,40) = 1.53, *p* = 0.22, *η* ^2^ = .03, such that the NK cell activity did not change overall with time (Fig. 6a). There was still a significant effect of group on NK cell activity, *F*(1, 40) = 3.14, *p* = 0.04, *η* ^2^ = .07, such that the NK cell activity of depressed hip fracture patients was reduced compared to healthy controls even 6 months after surgery. We also compared perforin expression in the NK cells of depressed hip fracture patients with and without depressive symptoms 6 weeks and 6 months post-surgery, but there was no significant effect of time, *F*(1, 13) = .27, *p* = 0.61, *η* ^2^ = .021 (Fig. 6b) such that perforin expression in NK cells did not improve even 6 months post-injury in depressed hip fracture patients.

## Discussion

Natural killer cells provide immune defence against viral infections and tumours (Cerwenka and Lanier 2001). An age-associated increase in NK cell number (Borrego et al. 1999; Sansoni et al. 1993) and CD56^dim/bright^ ratio (Chidrawar et al. 2006; Gayoso et al. 2011) alongside a decline in NK cell cytotoxicity has been well documented (Camous et al. 2012; Hazeldine et al. 2012). Additionally, numerous studies have reported a suppressive effect of severe life stressors such as caregiving (Irwin et al. 1987b), bereavement (Gerra et al. 2003; Irwin et al. 1988; Kiecolt-Glaser et al. 1987) and occupational stress (Boscolo 2009; Boscolo et al. 2009) on NK cell activity. Here, we report for the first time that the combination of hip fracture and depression results in further suppression of the age-related decline in NK cell activity. Crucially, our data show that hip fracture alone did not affect NK cell function, and it was only the addition of the psychological distress of depression that induced further loss of NK cell activity.

Our findings are consistent with previous reports of suppressed NK cell cytotoxicity in depressed individuals (Irwin et al. 1990; Jozuka et al. 2003; Kronfol et al. 1989; Maes et al. 1994), and the literature also supports our conclusion that this suppression in NK cell cytotoxicity in depressed patients is not a result of alterations in numbers of circulating NK cells (Darko et al. 1988; Maes et al. 1994) or a result of alterations in CD56^dim^/CD56^bright^ NK cell distribution or CD16^+ve^ NK cells. Interestingly, we have also found an association between depression scores and NK cell cytotoxicity in hip fracture patients, which has been reported in people undergoing severe life stressors, such as bereavement (Irwin et al. 1987a). Reduced NK cell cytotoxicity has been associated with an increased incidence and reduced survival during viral infections (Levy et al. 1991). Furthermore, epidemiological data has suggested an association between depressive symptoms and increased cancer morbidity and mortality (Shekelle et al. 1981). Taken together, these data suggest that the impairment in NK cell activity of depressed hip fracture patients might be of clinical significance and have implications for the health of these patients helping to explain in part the high 1-year mortality in this patient group (Nightingale et al. 2001).

Even though a number of studies have reported impaired NK cell activity on chronic exposure to psychological distress, none of these studies have tried to explain the reason behind this impaired killing by NK cells. In an attempt to examine the mechanism behind suppressed NK cell cytotoxicity, we first investigated target-effector conjugate formation and measured LFA expression (adhesion molecule) which was comparable between hip fracture patients with and without depression and healthy controls, suggesting that NK cell capacity to bind to target cells is not responsible for compromised NK cell cytotoxicity. These data contradict a previous report linking suppressed NK cell function in the family members of cancer patients with reduced conjugate formation (Montelli et al. 2001), but this may be a reflection of the ages of the two groups studied. When comparing other aspects of the NK cell cytotoxic process, we noted significantly lower perforin content in cells of depressed hip fracture patients. As far as we are aware, this is the first study to suggest that psychological distress-induced impairment in NKCC might be due to reduced perforin expression. The essential role of perforin in granule exocytosis and hence NKCC has been confirmed by studies in perforin-deficient mice, showing an increased susceptibility towards tumours (van den Broek et al. 1996) and inefficient control of viral challenges (van Dommelen et al. 2006) in these mice due to impairments in NKCC (Kagi et al. 1994). Interestingly, a study examining the effect of a psychosocial therapy on psychological responses reported a reduction in depression scores and also improved NK cell activity by increasing their perforin and granzyme expression (Li and Kawada 2011).

Our data suggested that elevated cortisol levels in the depressed hip fracture patients might be a contributing factor towards impaired NK cell cytotoxicity. Although raised circulating levels of cortisol did not mediate the effects of depression on NKCC, our in vitro analyses with dexamethasone suggest that cortisol is involved, although potentially contributing separately to both depressive symptoms and reduced NK cell function. In vitro studies have previously shown a suppressive effect of dexamethasone on killing by an NK cell line (Bush et al. 2012; Callewaert et al. 1991; Krukowski et al. 2011) and peripheral blood leukocytes enriched for NK cells (Holbrook et al. 1983). Here, we confirm that a physiologically equivalent dose of dexamethasone also suppressed NKCC in isolated human NK cells. Interestingly, a clinical study has reported suppressed NK cell activity in healthy subjects following an infusion of cortisol, epinephrine and glucagon to mimic the response to burn injury (Blazar et al. 1986). In addition, we show a reduction in serum DHEAS levels in the depressed hip fracture patients. Previous findings have reported a positive effect of DHEAS on NK cell cytotoxicity (Solerte et al. 1999), though we did not find an association between serum DHEAS and NKCC. Taken together, our data and the published literature suggest that increased HPA axis activity in depressed hip fracture patients is contributing to suppressed NK cell activity in these patients, although not directly mediating the association between depression and NK cell function.

In vitro dexamethasone experiments add further support for a role of increased HPA axis activity in reduced NKCC. Reduced intracellular perforin expression was found here and was also reported previously in NK cells treated with dexamethasone (Bush et al. 2012). Furthermore, Mifepristone (a glucocorticoid antagonist) can augment NK cell cytotoxicity via increased perforin expression mediated by ERK activation, which can be blocked by cortisol (Qian et al. 2012). These reports support our proposal that prolonged exposure of NK cells to cortisol lowers the expression of perforin, resulting in a reduced ability of NK cells to induce target cell lysis. It is possible that high cortisol separately results in increased depressive symptoms rather than vice versa, which would explain why depressive symptoms, cortisol and NK cell function are all related, but cortisol level does not statistically mediate the association between depression and reduced NK cell function. In an observational study such as this, we are unable to determine the direction of causality.

Although we were unable to investigate the molecular mechanisms underlying suppressed perforin expression in the NK cells of depressed individuals with higher serum cortisol levels, previous studies have suggested that glucocorticoids induce epigenetic modifications and regulate gene transcription of immune responsive genes (Mifsud et al. 2011). Glucocorticoids induce reduced acetylation at the histone 4 lysine 8 (H4-K8) position, possibly resulting in reduced expression of genes such as perforin (Krukowski et al. 2011).

In this study, we have linked psychological distress with HPA axis activation and suppressed NK cell cytotoxicity. However, the HPA axis is not the sole pathway mediating stress-related immune suppressive effects. For instance, a study reporting an elevated cortisol level and suppressed NK cell activity in bereaved women compared to controls also reported a significant reduction in NK cell activity in women anticipating bereavement without elevated cortisol levels (Irwin et al. 1988). Further, in our own work on neutrophil function, depressed patients again had the highest cortisol level and poorest neutrophil function, but cortisol did not mediate the relationship between depressive symptoms and neutrophil superoxide production (Duggal et al. 2013). Depression has been associated with sympathetic nervous system activation and release of neuropeptides (epinephrine, neuropeptide Y), which has a suppressive effect on NK cell activity (Irwin et al. 1991; Maes et al. 1994) by reducing levels of perforin and granzyme B (Dokur et al. 2004). Another cortisol-independent mechanism via opioid peptide release on exposure to stress is known for reducing NK cell cytotoxicity (Shavit et al. 1984).

Stress-induced immune dysregulation may persist even after chronic stress has ablated (Kiecolt-Glaser et al. 1996). Hip fracture is associated with an increase in mortality that can persist for years after the fracture. Recovery after hip fracture is a long process, and only one third of patients have been reported to return to their pre-fracture functional status 1 year after the fracture (Magaziner et al. 2000). We have reported that depression results in a long-term suppression of NK cell functioning in hip fracture patients. Additionally, we have also reported that perforin expression in depressed hip fracture patients did not improve even 6 months post-surgery.

In conclusion, in this study, we propose that the development of depression in hip fracture patients results in long-term impairment in their NK cell functioning. Our results suggest that therapy to reduce the incidence of depression in these patients could not only improve the quality of life in the elderly after hip fracture but also reduce risk of life-threatening infections.

## Materials and methods

### Participants

One hundred one hip fracture patients were recruited from five hospitals in Birmingham, UK, between 2010 and 2012. Inclusion criteria were that participants had to be aged 60 years and over with a hip fracture sustained 4–6 weeks previously but with no chronic immune disorders or taking any regular medications that might modify immunity. Additionally, patients must not have had a previous history of chronic depression. Fifty healthy older adults were also recruited from the community as controls. The study was approved by South Birmingham Local Research Ethics Committee, and all participants provided written informed consent (study ref: 09/H1203/80).

### Study design

The study was a prospective case-control design with three groups of older adults: hip fracture patients with or without depression and healthy older adults. Consent was gained whilst patients were still in hospital. All participants completed questionnaires, structured interviews and provided a blood sample (40 ml) 4–6 weeks and 6 months after hip fracture. The blood sample was used for a broad range of immune function tests, and for this reason, only 10 ml was available to NK cell analysis. Thus, it was not always possible to gain sufficient cells for all analyses, and the sample size is therefore shown in each figure legend. Blood samples were taken between 0900 and 1100 hours to minimise any effect of diurnal variations in steroid levels or immune function. None of the patients had an infection at the time of blood sampling. Assays for NK cell cytotoxicity were performed on the same day as blood sampling; remaining PBMCs were frozen down for later NK cell phenotypic analysis. Serum was frozen for subsequent analysis of adrenal hormone levels.

### Assessment for depressive symptoms

Standard socio-demographic and health behaviour information were taken, and all medications, prescription and over-the-counter were recorded by the interviewer. The psychological status of the participant was assessed by means of standardised psychometric questionnaires. The presence of depressive symptomatology was evaluated using a Geriatric Depression Scale (Yesavage et al. 1982), and depression was defined as a GDS score greater than or equal to 6 on this scale.

### Immunostaining for phenotypic analysis of NK cells

Peripheral blood mononuclear cells (PBMCs) were isolated by density centrifugation using Ficoll-Paque™ PLUS (GE Healthcare, Uppsala, Sweden). PBMCs were frozen down by re-suspending cells in freezing medium consisting of 10 % DMSO (Sigma-Aldrich, UK) in heat-inactivated fetal calf serum (Biosera, UK) and transferring them in small aliquots into cryovials. The cryovials were transferred into a freezing container (Mr Frosty, Sigma-Aldrich, UK) containing isopropanol (VWR International, UK), and the frozen cells were then stored at −80 °C. Frozen samples were thawed in a water bath at 37 °C and washed in RPMI 1640. PBMCs were then resuspended in phosphate-buffered saline (PBS) at a concentration of 1 × 10^6^/ml and were stained with anti-human CD3-FITC (Dako Ltd, Cambridge, UK; clone UCHT1), CD56-PE (Dako Ltd; clone C5.9), CD11a-FITC (Dako Ltd; clone MHM24), CD16-FITC (eBiosciences, Hatfield, UK; clone CB16), CD57-FITC (BioLegend, London, UK; HCD57) or NKG2D-PEcy7 (BioLegend; 1D11) for 20 min at 4 °C. Post-incubation, cells were washed and resuspended in PBS and transferred into FACS tubes for flow cytometric analysis using a Cyan™ ADP flow cytometer (Dako Ltd). Appropriate isotype controls were used for setting of gates. The CD56^dim^ to CD56^bright^ ratio was determined on absolute numbers of cells.

### NK cell cytotoxicity

NK cells were isolated from PBMCs by negative selection using MACS technology (Human NK cell isolation kit; Miltenyi Biotech, Germany), and purity of isolated NK cells was assessed by immunostaining for CD56 and flow cytometry. Purity obtained on a routine basis was >92 %. The MHC class I-deficient NK-sensitive cell line K562 was used as the target population for NK cell cytotoxicity (NKCC) assays. K562 cells were obtained from the American Type Culture Collection (ATCC, Middlesex, UK; ATCC number CCL-243) and were maintained in medium containing 2 mM l-glutamine, 100 U/ml penicillin and 100 μg/ml streptomycin (Sigma-Aldrich, Poole, UK) supplemented with 10 % heat-inactivated fetal calf serum (Sera Laboratories International, Sussex, UK) at 37 °C in a humidified 5 % CO_2_ atmosphere. Cells were split one in three on the day preceding the assay. NKCC was assessed by two-colour flow cytometry using an adapted version of the protocol described by Godoy-Ramirez and colleagues (Godoy-Ramirez et al. 2000). Briefly, NK cells and K562 cells in a final effector (E) to target (T) cell ratio of 10:1 were incubated together in a 96-well U bottom plate at 37 °C in a humidified 5 % CO_2_ atmosphere for 2 h. Due to the limited amount of blood available from hip fracture patients, only one E/T ratio (10:1) could be performed, though the assay initially validated over a range of E/T ratios (20:1, 10:1, 5:1 and 2.5:1) and incubation times to establish 2 h as an optimal time point. To account for spontaneous lysis of target cells during this period, K562 cells were incubated in the absence of effector cells. Post-incubation, cells were pelleted and stained with antibody CD56-PE (Dako Ltd; clone C5.9). After 20 min of labelling on ice, cells were washed once in PBS, and the subsequent pellet was re-suspended in PBS. To detect cell death, sytox blue cell stain (pre-diluted 1:800 in PBS; Invitrogen, Paisley, UK) was added to each well, and the cells were transferred to FACS tubes and samples analysed by flow cytometry using a Cyan™ ADP flow cytometer (Dako Ltd). To assess the percentage of lysed target cells within the sample, NK cells and K562 cells were distinguished from one another via staining using anti-CD56 antibody. The cytometry gate was set on K562 target cells to exclude effector (NK cells) (Fig. 1a), and target cell death was measured. Results were expressed as % specific lysis and calculated by the formula:

$$ \%\;\mathrm{Specific}\ \mathrm{lysis}=\left[\left(\mathrm{test}-\mathrm{spontaneous}\right)/2000\right]\times 100 $$

Test represents the percentage of target cells lysed by NK cells; spontaneous represents the percentage of target cells lysed in culture in the absence of NK cells. Post-incubation, immunophenotyping and scatter profiles were used to distinguish between effector (NK cells), target cells (K562 cells) and conjugates of both cells.

### Measurement of perforin and granzyme B expression in NK cells

PBMCs (1 × 10^6^/ml) were stained for extracellular surface markers to identify NK cells CD3-FITC (Dako Ltd; clone UCHT1) and PE-conjugated CD56 (Dako Ltd; clone C5.9) for 20 min in the dark at 4 °C. Cells were washed with PBS and re-suspended in 50 μl of Reagent A (Fix and Perm kit, Invitrogen) and incubated for 30 min in the dark at 20 °C. Post-fixing, cells were then washed with PBS and centrifuged at 250×*g* for 5 min. The pelleted cells were resuspended in 50 μl of Reagent B (Fix and Perm kit, Invitrogen) and were stained with anti-human Perforin-FITC antibody (BioLegend; clone: Dg9) or with anti-human granzyme-FITC antibody (BioLegend; clone: GB11) for 30 min in the dark at 20 °C. Finally, the cells were washed and resuspended in PBS and analysed by flow cytometry (Cyan™ ADP, Dako). Appropriate isotype controls were used for gate setting.

### Serum cortisol and DHEAS assays

Serum cortisol and DHEAS levels were measured by ELISA using a commercial kit (IBL international, Hamburg, Germany) according to the manufacturer’s instructions. Intra-assay coefficients of variation (CV %) were 6.7 for cortisol and 4.6 for DHEAS ELISAs.

### In vitro dexamethasone treatment of NK cells

NK cells isolated (1 × 10^6^ cells/ml) from young donors were incubated in 96-well round bottomed plates in the presence of water soluble dexamethasone (Sigma-Aldrich) at 10^−5^-, 10^−7^- and 10^−9^-M concentrations or distilled water (control) for 18 h. The physiologically relevant concentration of dexamethasone approximates to 10^−7^ M (Bush et al. 2012; Krukowski et al. 2011). Post-incubation, cells were washed twice with RPMI 1640 medium (Sigma-Aldrich) and NK cells were resuspended to 1 × 10^6^ cells/ml for further analysis.

### Annexin V staining to measure apoptosis

Annexin V binds to phosphatidylserine exposed on the outer leaflet of apoptosis cells and can thus be used to identify apoptotic cells (Andree et al. 1990). Isolated NK cells (1 × 10^6^) were resuspended in 1× Annexin V Binding buffer (BD Biosciences, UK). Annexin V-FITC (BD Biosciences, Oxford, UK) was added to the cells, and after gentle vortexing, the cells were incubated for 10 min at 4 °C in the dark. Post-staining, the cells were then transferred into a FACS tube containing 1× Annexin V Binding buffer and were analysed for Annexin V binding by flow cytometry (Cyan™ ADP, Dako). NK cell death was also measured by immunostaining isolated NK cells (1 × 10^6^) resuspended in 100 μl of PBS with 10 μl of sytox blue cell stain (pre-diluted 1:800 in PBS; Invitrogen) followed by analysis via flow cytometry.

### Assessing NK cell activation status

NK cell activation was assessed by measuring expression of CD69 and the degranulation marker CD107a. Isolated NK cells (1 × 10^6^/ml) were incubated with K562 cells (1 × 10^5^/ml) in a final effector (E) to target (T) cell ratio of 10:1 at 37 °C in a humidified 5 % CO_2_ atmosphere for 2 h. Post-incubation, cells were washed and re-suspended in PBS and immunostained using anti-human CD56-PE antibody (Dako; clone C5.9) and anti-human CD69-FITC antibody (eBiosciences; clone FN50) on ice for 20 min in the dark. After which, cells were washed and resuspended in PBS and analysed for CD69 positivity by flow cytometry (Cyan™ ADP, Dako). The percentage of CD69 expressed by 4000 NK cells was recorded.

Granule fusion with the NK cell membrane was assessed using a slightly modified version of a CD107a degranulation assay previously described by Alter and colleagues (Alter et al. 2004). PBMCs (1 × 10^6^/ml) were incubated with K562 cells at an E/T ratio of 1:1 in the presence of 5 μl of anti-CD107a-FITC antibody (eBiosciences; clone: eBio H4A3) for 1 h at 37 °C in a humidified 5 % CO_2_ atmosphere. After 1-h incubation, 6 μg/ml of monensin (Sigma-Aldrich) was added, and the samples were incubated for a further 2 h. NK cells (1 × 10^6^/ ml) incubated alone served as controls. Post-incubation, the cells were pelleted and resuspended in PBS and stained with anti-human CD56-PE antibody (Dako Ltd; clone C5.9) and anti-human CD3-Pacific blue antibody (BD biosciences; clone: UCHT1) for 20 min at 4 °C in the dark. After which, co-cultured cells were washed and resuspended in PBS, and CD107a expression on 4000 NK cells was recorded by flow cytometry.

### Statistical analysis

Univariate ANOVA with least significant difference post hoc tests was used to assess differences between the three groups. Where demographic variables differed significantly between the groups, analyses were rerun adjusting for these variables using ANCOVA. Pearson’s correlations were used to examine associations between depression score and NK cell function and stress hormone levels. In order to test for potential mediation between depression group and immune outcomes by stress hormones, a series of linear regression models were run. Group (depressed hip fracture, non-depressed hip fracture, healthy controls) was entered into the model at step 1 with the immune outcome as the dependent variable. This was then repeated with the relevant stress hormone entered at step 2, to examine effects on the original associations between group and immune outcome. Where any of these original associations became non-significant after entering the potential mediator (stress hormone), mediation was deemed to have occurred and checked using the Sobel test.
